# Supplementary material for: Pain catastrophizing as a mediator of the relationship between pain intensity and depression: Evidence from chronic pain patients in Gaza
Source: Glob Ment Health (Camb). 2026 Feb 11;13:e39. doi: 10.1017/gmh.2026.10151 (PMC12973239; doi:10.1017/gmh.2026.10151)

**Supplementary Figure S1**. Scatterplot showing the correlation between pain intensity (NRS) and pain catastrophizing (PCS) scores (rs = 0.39, p < 0.001). Each point represents an individual participant (n = 272). The blue line represents the linear regression fit with 95% confidence interval (gray shading).

**Supplementary Figure S2.** Scatterplots showing correlations between pain intensity (NRS) and psychological distress measures from the DASS-21. (A) Depression subscale (rs = 0.29, p < 0.001); (B) Anxiety subscale (rs = 0.22, p < 0.001); (C) Stress subscale (rs = 0.31, p < 0.001). Each point represents an individual participant (n = 272). Blue lines represent linear regression fits with 95% confidence intervals (gray shading).


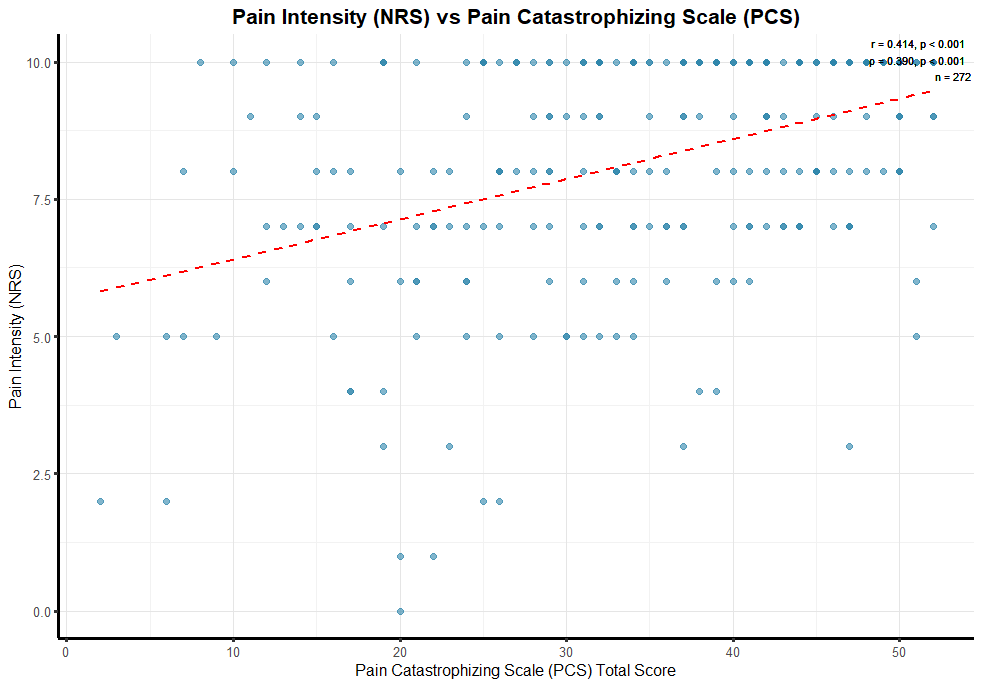


**Supplementary Figure 1:** Simple scatter plot of Pain Catastrophizing Scale (PCS) score by Pain Intensity (NRS).


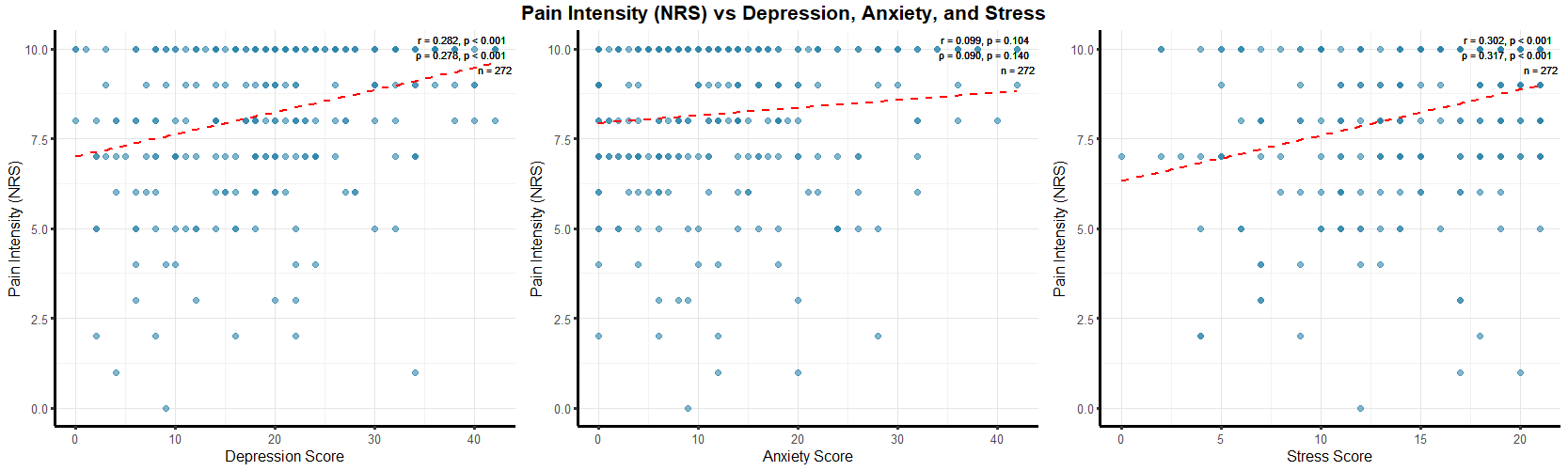


**Supplementary Figure 2:** Simple scatter plot of DASS-21 Subscales by Pain Intensity (NRS).

**Supplementary Figure S3.** Item-level correlations between pain intensity (NRS) and DASS-21 Depression subscale items. The seven depression items assess dysphoria, hopelessness, devaluation of life, self-deprecation, lack of interest/involvement, anhedonia, and inertia (items 3, 5, 10, 13, 16, 17, 21). Each panel shows the Spearman correlation coefficient (rs) and linear regression fit with 95% confidence interval. Points represent individual participants (n = 272).


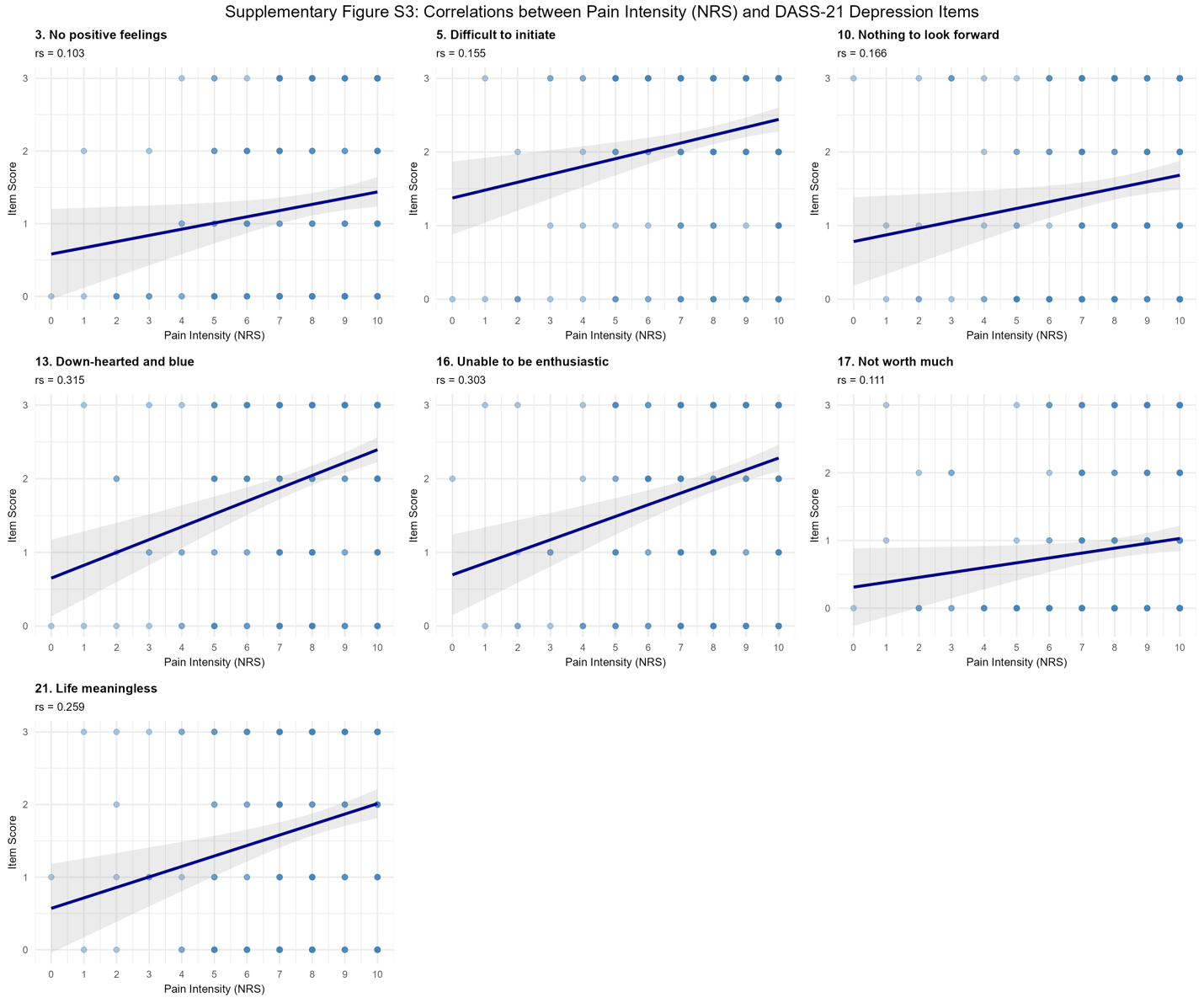


**Supplementary Figure S4.** Item-level correlations between pain intensity (NRS) and DASS-21 Anxiety subscale items. The seven anxiety items assess autonomic arousal, skeletal muscle effects, situational anxiety, and subjective experience of anxious affect (items 2, 4, 7, 9, 15, 19, 20). Each panel shows the Spearman correlation coefficient (rs) and linear regression fit with 95% confidence interval. Points represent individual participants (n = 272).


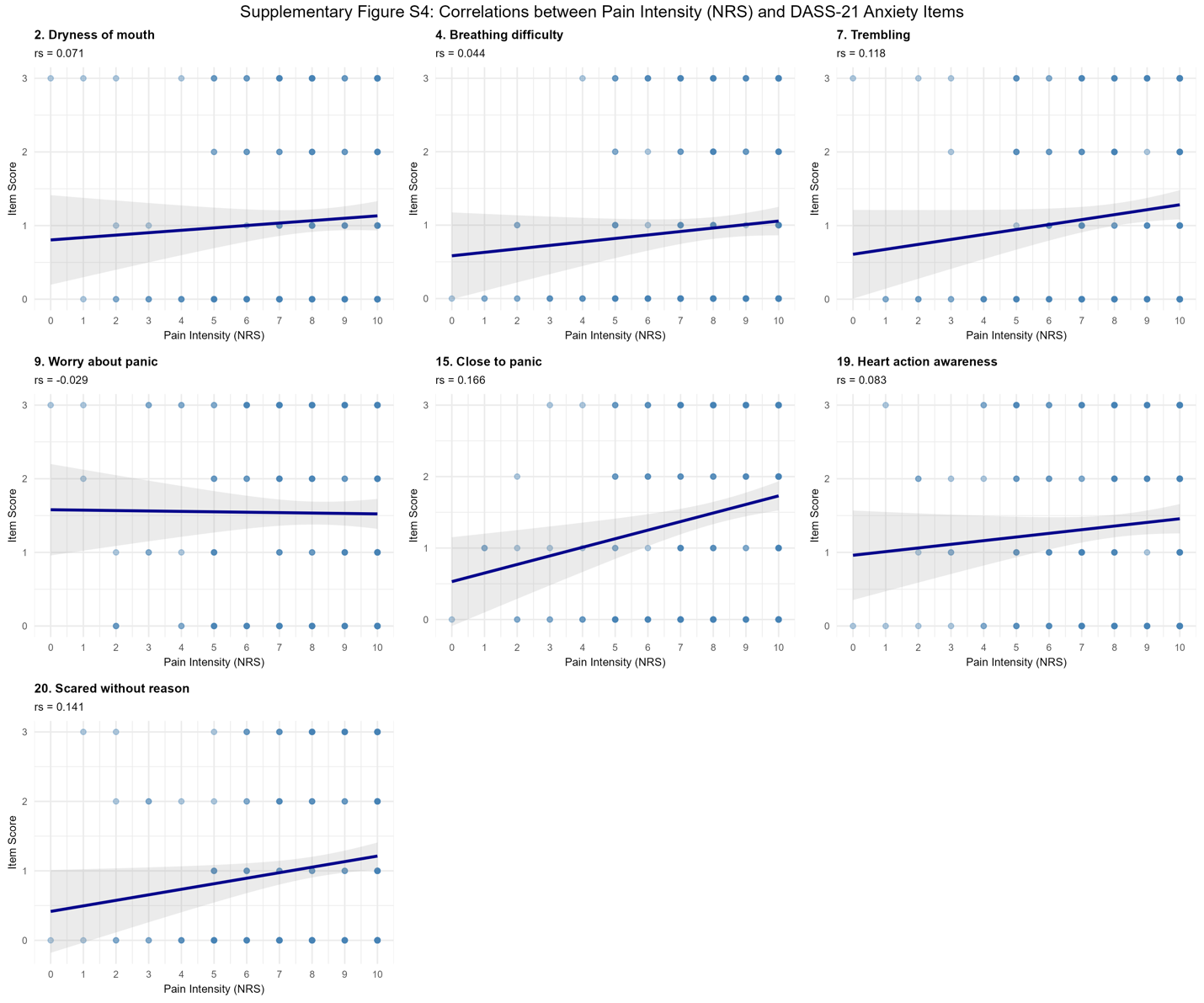


**Supplementary Figure S5.** Item-level correlations between pain intensity (NRS) and DASS-21 Stress subscale items. The seven stress items assess difficulty relaxing, nervous arousal, being easily upset/agitated, irritable/over-reactive, and impatient (items 1, 6, 8, 11, 12, 14, 18). Each panel shows the Spearman correlation coefficient (rs) and linear regression fit with 95% confidence interval. Points represent individual participants (n = 272).


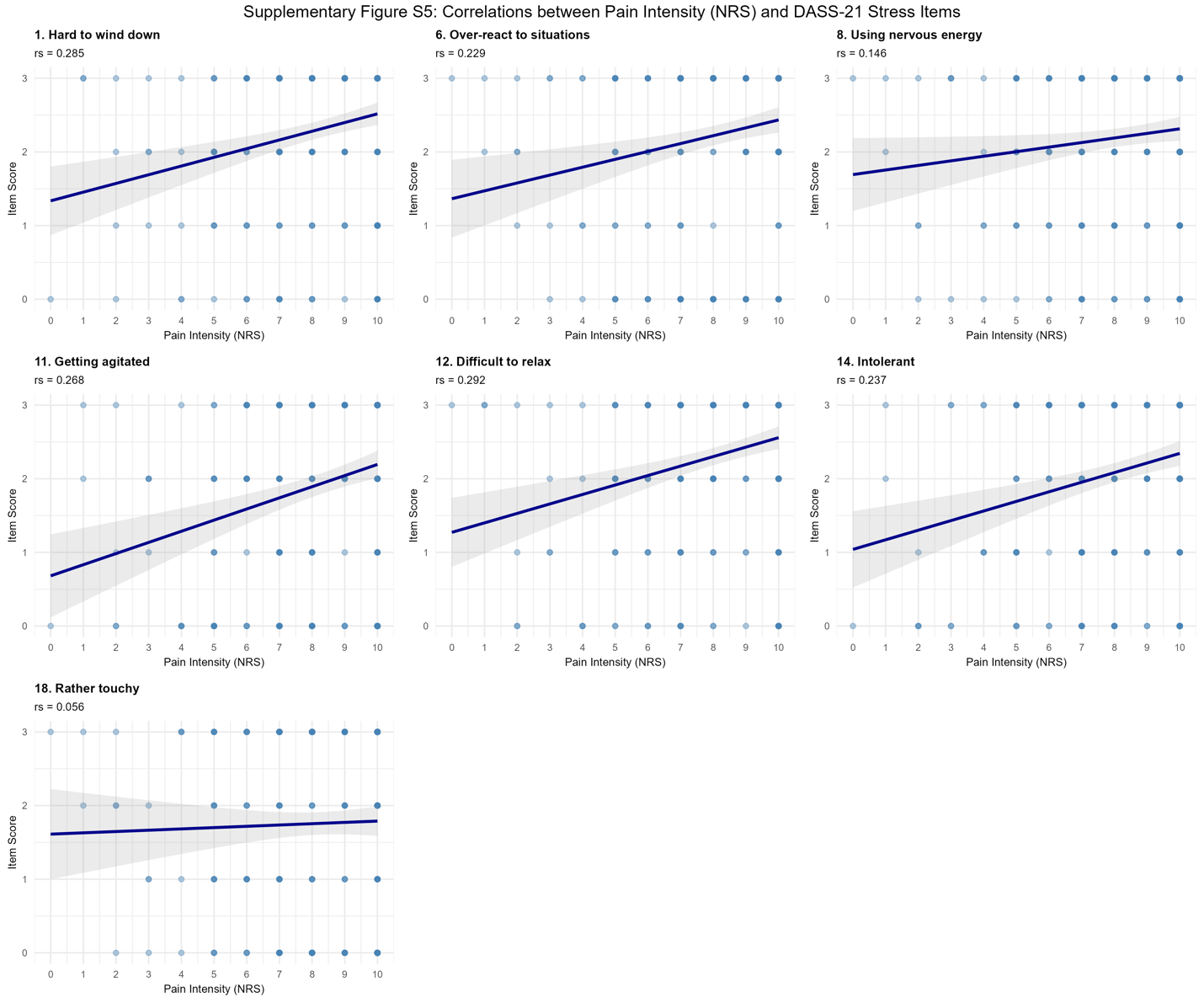

Supplement: AbuJlambo et al. supplementary material [file S2054425126101514sup001.docx]
